# New editor at the most exciting time in tobacco control

**DOI:** 10.1136/tc-2023-058413

**Published:** 2023-10-19

**Authors:** Ruth E Malone

**Affiliations:** Social and Behavioral Sciences, University of California, San Francisco, San Francisco, California, USA

**Keywords:** Advocacy, Policy, Endgame

When I was appointed the third Editor-in-Chief of *Tobacco Control* in 2009, I did not foresee that the new role I was about to undertake would become a high point of my career. I did not yet completely realise the abundant opportunities I would have to help junior and less-experienced authors develop and publish their work, to curate the scientific and analytic literature that influences the field, and to call out and opine on emerging and contested issues in the field. Now, after many joyous, vexing, overloaded and exciting years of editing, I am ready to step down, leaving the top specialty journal in the field in capable hands with longtime Senior Editor and previous News Editor Dr Marita Hefler appointed as the journal’s new Editor-In-Chief.

A journal’s success is never attributable to any one person. I am so grateful to all the senior editors with whom I have been fortunate to work and to all the sub-editors serving in various capacities over the years. I have benefited from the unwavering support of the Editorial Advisory Board and the publisher, *BMJ Journals*. I am particularly thankful to our reviewers, whose expertise and guidance to the editors helps us to produce a quality journal. Most of all, I appreciate our authors and readers.

The year 2009 was an exciting time in tobacco control. In the USA, legal cases and whistleblower actions had resulted in the release of millions of pages of internal tobacco company documents, which I and many others were researching as fast as we could comb through them. During the preceding years (in a development partly spurred by the revelations in the industry documents about how the tobacco industry had worked to undermine the WHO), the WHO Framework Convention on Tobacco Control had become one of the most rapidly embraced treaties in history, galvanising the development of a global network of NGOs that pressured governments to pass effective policy measures and reduce tobacco’s toll of preventable disease and premature death. Smokefree laws were dramatically reshaping the landscape of tobacco use. Today, it seems unimaginable that we once accepted smoking in elevators, hospitals, day care centres and airplanes.

Later in 2009, the US Food and Drug Administration was at last given the authority to regulate tobacco products for the protection of the public health, with many optimistic that the agency would finally reduce nicotine in cigarettes to non-addictive levels, as advocates had been urging for at least a decade. Doing so would increase the likelihood that the ‘choice’ to smoke endlessly postulated publicly by tobacco companies (while privately, they engineered cigarettes to enhance their addictiveness) really could perhaps be freely made. But nicotine is the ‘lifeblood’ of the industry,^1^ and regrettably, it remains so. To date, the USA still has mostly only good intentions to show for its federal-level regulatory efforts, with millions more lives wastefully sacrificed to tobacco industry profits in the intervening years. We continue to hope for nicotine regulation and the removal of menthol from tobacco products.

Meanwhile, the industry moved into the emerging e-cigarette market with such enthusiasm that—just as youth smoking rates were finally dropping—a whole new generation of youth became hooked on nicotine. In the USA, JUUL (acquired by Altria, the largest tobacco company in the USA) was marketed in ads reminiscent of old cigarette ads, featuring youthful models having so much fun, with predictable results: 14% of high school students reported current vaping in 2022, with more than 4 out of 10 vaping on 20 out of the last 30 days.^2^ Globally, some countries encouraged the use of e-cigarettes as potentially less harmful products; some banned them due to the paucity of evidence on potential long-term risks and benefits; and the divisions within the tobacco control movement about what to do about the endlessly proliferating range of these non-combusted cigarette, non-cigarette, tobacco, non-tobacco, nicotine, non-nicotine, menthol and non-menthol products were gleefully exploited by tobacco companies, consistent with their long-term plans to achieve ‘normalisation’.

Our current moment in tobacco control can be characterised as a struggle between two competing narratives: (a) tobacco use is a problem of unhealthy individual choices that can only be remedied by offering more choices, a recycled tobacco industry favourite; and (b) the endgame story—tobacco use is a problem of institutional failures to protect the public, highlighting increasingly absurd and unsustainable contradictions.

We cannot continue forever to say that cigarettes kill but still sanction sales of the single most deadly consumer product in history as though it is normal to sell death. We cannot continue to claim we care about protecting public health while staying quiet as government agencies whose mission is to protect the public are grotesquely captured or hamstrung by industry influence. We cannot forever expand the often-ineffective age-based restrictions on purchasing products as though the products were only harmful to the young but benign or useful to older people. We cannot continue to endlessly study and develop new policies to limit harmful chemical constituents about which the industry always knows far more than we do while new products flood the market at an unprecedented rate.

Regardless of how one feels about the potential of newer products to contribute to true ‘harm reduction’, we must make governments develop plans to end commercial cigarette sales. It will not happen all at once or overnight. There will be problems that must be thought through. But we cannot do it without talking about it and developing concrete plans—and we cannot, must not, continue to accept the status quo of this industrially produced and perpetuated epidemic.

The most exciting time in tobacco control is always now. Despite the ‘two steps forward, one step back’ process that has long characterised our field of battle, some countries and jurisdictions have managed to beat back Big Tobacco’s influence sufficiently that serious conversations about planning for an endgame for the tobacco epidemic are taking place. Five years ago in this journal, I predicted: ‘Within ten years, some countries in the world (or states in the USA) will have begun a staged, systematic phaseout of the sale of commercial cigarettes and/or other tobacco products’.^3^

Now, in less than half that time, it is starting to happen. Endgame is a worldwide conversation being furthered by substantive policy change. In New Zealand, a comprehensive set of policy measures^4^ including mandated nicotine reduction for cigarettes, large reductions in retail availability and ending sales to anyone born after a specific date (the ‘Tobacco-Free Generation’ idea, first proposed in this journal^5^ and also enacted in some Massachusetts cities)^6^ is being rolled out. In California, the US state with the second lowest smoking prevalence, a statewide ban on sales of flavoured tobacco products survived an initiative challenge from the tobacco industry^7^; two California cities have banned sales of tobacco products, and others have ended tobacco sales in certain types of outlets, including pharmacies. The Netherlands plans to reduce tobacco retail outlets through a licensing scheme.^8^ Numerous countries, including Finland, Scotland, Ireland, England, Sweden and Canada, have set endgame goals. Smokefree policies have continued to expand. Much of the research and evidence underpinning these policy measures^9^ first appeared in this journal.

The public is more ready than many assume. Approximately 70% of people who smoke consistently say they want to quit,^10^ and they almost universally regret starting.^11^ More than half the respondents in a recent US CDC-sponsored study supported a policy prohibiting the sale of tobacco products.^12^ Given that no public campaign has ever previously sought to convince people that this would be a good thing to do, it is clear that baseline support for phasing out sales is high. The contradictions are simply becoming too great to sustain.

Will achieving the tobacco endgame mean no one will ever smoke or use tobacco? No. It means that we will end the deeply malign influence of the tobacco industry on our society, and gradually, its shocking contribution to premature disease and death will subside. The industry is powerful, but not as powerful as it once was. Thirty years hence, those who come after us will wonder why for so many decades we tolerated this industry, as millions fell by the wayside.

The greatest question of our time is whether we will in the end sacrifice everything—our children’s lives, our future health, even the planet that supports all life—to corporate profits. Tobacco is of a piece with the other issues that now constitute the greatest threat to societal well-being. As the work goes on, we must stay focused on advancing the endgame narrative through concrete systems planning and never allow disagreements to cause us to lose sight of the goal: reaching together a time when we end this epidemic for good.
